# Characterization of Synthetic Hydroxyapatite Fibers Using High-Resolution, Polarized Raman Spectroscopy

**DOI:** 10.1177/0003702820942540

**Published:** 2020-07-28

**Authors:** Furqan A. Shah

**Affiliations:** Department of Biomaterials, Sahlgrenska Academy, University of Gothenburg, Gothenburg, Sweden

**Keywords:** Hydroxyapatite, calcium phosphate, Raman spectroscopy, biomaterials, bone

## Abstract

In the Raman spectrum of B-type carbonated apatites, the ν_1_ CO_3_^2–^ mode (at ∼1070 cm^–1^) overlaps the ν_3_ PO_4_^3–^ band. The latter is readily observed where the CO_3_^2–^ content is low (up to ∼3 wt%). The CO_3_^2–^ content of bone is considerably higher (∼7–9 wt%). As a result, the ν_3_ PO_4_^3–^ band becomes completely obscured. The 1000–1100 cm^–1^ spectral range of carbonated apatite is frequently considered a combined ν_3_ PO_4_^3–^ and ν_1_ CO_3_^2–^ region. Here, high-resolution polarized Raman spectroscopy (step size of 0.74 ± 0.04 cm^–1^) provides new insights into synthetic hydroxyapatite (HAp) obtained as micrometer-sized fibers. Compared to bone mineral (deproteinized bovine bone), spectral features of HAp fibers are highly resolved. In particular, the ν_3_ PO_4_^3–^ band resolves into nine distinct sub-components: 1028, 1032, 1040, 1043, 1047, 1053, 1055, 1062, and 1076 cm^–1^. Parameters including full width half-maximum, intensity, area fraction, intensity ratio, and area fraction ratio vary between parallel and perpendicular polarized configurations. It is likely that the ν_1_ CO_3_^2–^ band of B-type carbonated apatites may contain a small but not insignificant contribution from the 1076 cm^–1^ sub-component of the ν_3_ PO_4_^3–^ band. Furthermore, the 1076 cm^–1^/1047 cm^–1^ ratio changes between parallel and perpendicular scattering configurations, suggesting that the contribution of the 1076 cm^–1^ sub-component may vary as a function of local orientation of bone mineral, thus skewing the ν_1_ CO_3_^2–^ band and compromising accurate estimation of carbonate-to-phosphate ratios in B-type CO_3_^2–^ substituted apatite.

## Introduction

Synthetic- and natural-derived calcium phosphates, e.g., hydroxy- or hydroxy(l)apatite (HAp), are by far the most frequently used biomaterials for bone repair.^1^ Naturally occurring (geological and biological) apatites can incorporate a plethora of anionic, cationic, and anionic complex substitutions.^2^ Likewise, ion substitutions can be easily achieved in synthetic apatites in order to tailor chemical stability or degradation behavior, and bone/biological response.^3^ Raman spectroscopy is frequently employed for nondestructive assessment of bone quality,^4,5^ and has been used extensively to study carbonate substitution in bone mineral,^6^ which is considered an important marker of bone turnover.^7^

In a typical Raman spectrum of B-type carbonated apatites (where CO_3_^2–^ substitutes for PO_4_^3–^), the symmetric stretching ν_1_ CO_3_^2–^ (b_2g_) mode overlaps the antisymmetric stretching ν_3_ PO_4_^3–^ (a_1g_) band. The latter is observed up to ∼3 wt% CO_3_^2–^ but tends to be completely enveloped by the ν_1_ CO_3_^2–^ peak in bone,^8^ where the CO_3_^2–^ content is significantly higher (∼7–9 wt%).^9^ CO_3_^2–^ substitution for PO_4_^3–^ influences physical properties including crystallite size, solubility, and thermal stability of biological apatites,^10^ in effect restricting mineral crystallinity to below that observed for carbonate-free apatites.

Polarized Raman spectroscopy has been used previously to investigate orientation dependence of bone mineral.^11–13^ Attributed to A- and B-type ν_1_ CO_3_^2–^ bands, previous investigations of scattering configuration dependence of the ν_3_ PO_4_^3–^ mode in carbonated fluorapatite have been unsuccessful in precisely locating the positions of individual sub-components.^14^ Using high-resolution polarized Raman spectroscopy, this work investigates micrometer-sized fibers of carbonate-free synthetic HAp and carbonated apatite of bovine cortical bone to better understand the ν_3_ PO_4_^3–^ and ν_1_ CO_3_^2–^ overlap in B-type CO_3_^2–^ substituted apatite such as bone mineral in order to enable more accurate estimation and interpretation of carbonate-to-phosphate ratios.

## Materials and Methods

### Synthetic HAp Fibers and Bovine Bone Mineral

The HAp fibers were obtained by dispersing 5–80 nm apatite particles in an aqueous solution of 200 kDa pullulan (Sigma-Aldrich). This solution was extruded at 1.4 kg/cm^2^ pressure while simultaneously discharging air at 250 m/s to form a stream of fibers. Using a far-infrared heater under the extrusion nozzles, the fiber stream was heated at 400 ℃ and blown onto a screen conveyor belt to produce a non-woven fabric that was further heated at a rate of 50 ℃ per hour and calcined at 1100 ℃ for one hour.^15^ Commercially sourced (https://boneslices.com) bovine cortical bone stored in 96% ethanol was deproteinized using 5% sodium hypochlorite (Honeywell Fluka) for 16 h at 4 ℃ in order to isolate the inorganic/mineral phase.

### Scanning Electron Microscopy and Micro-Raman Spectroscopy

The HAp fibers were visualized using scanning electron microscopy (Ultra 55 FEG SEM, Leo Electron Microscopy Ltd, UK) in the secondary electron mode. Micro-Raman spectroscopy was performed using a confocal Raman microscope (Renishaw inVia Qontor) equipped with a 633 nm laser and LiveTrack focus-tracking technology. The laser was focused down on to the sample surface using a × 100 (0.9 NA) objective. In the 390–1100 cm^–1^ spectral range (step size of 0.81 cm^–1^ at 390 cm^–1^ and 0.68 cm^–1^ at 1100 cm^–1^), the Raman scattered light was collected using a Peltier-cooled charge-coupled device deep depletion near-infrared-enhanced detector behind a 2400 g mm^–1^ grating, 10 s integration time, and 10 accumulations. Using a half-wave plate and polarization analyzer, spectra were collected in parallel $\text{z}(\mathrm{xx})\bar{\text{z}}$ and perpendicular $\text{z}(\mathrm{xy})\bar{\text{z}}$ polarized configurations from 3 × 3 point grid-matrices (*n* = 8) for HAp fibers and from 9 × 8 point grid-matrices (*n* = 1) for deproteinized bovine bone. The laser power at the sample was *∼*15 mW. Background subtraction and cosmic ray removal were performed in Renishaw WiRE 5 software. Spectra from each grid-matrix were averaged and normalized to the 428 cm^–1^ ν_2_ PO_4_^3–^ sub-component. For HAp fibers, one Lorentzian curve was fitted to the ν_1_ PO_4_^3–^ peak (*∼*962 ± 10 cm^–1^). Centered at inflection points in calculated second-derivative spectra, ν_3_ PO_4_^3–^ band (1020–1090 cm^–1^) sub-components were resolved by fitting multiple Lorentzian curves (*r*^2 ^> 0.99). For each sub-component, the full width half-maximum (FWHM), intensity, and area fraction of the fitted range were obtained. Polarization configuration dependent variation in intensity ratios and area fraction ratios between ν_3_ PO_4_^3–^ sub-components were expressed as percentage difference values.

### Statistical Analysis

For statistical analysis, the non-parametric Wilcoxon signed-rank test was used (SPSS Statistics, v.25, IBM Corporation) and *p* values < 0.05 were considered statistically significant. Mean values ± standard deviations are presented.

## Results and Discussion

**Figure 1.**
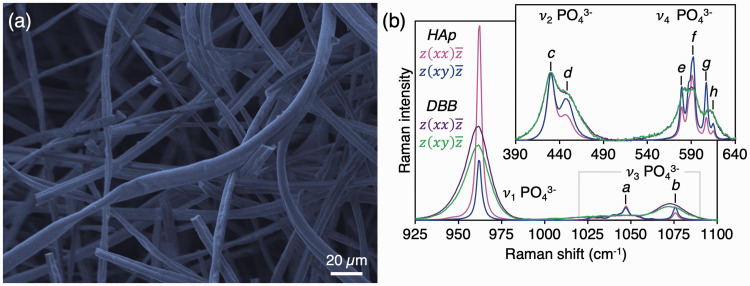
(a) Micrometer-sized fibers of synthetic hydroxyapatite (HAp) observed using scanning electron microscopy. (b) The ν_1_ PO_4_^3−^ and ν_3_ PO_4_^3−^ bands in HAp fibers (magenta and blue) and deproteinised bovine bone (DBB; purple and green). Average Raman spectra normalized to the 428 cm^−1^ ν_2_ PO_4_^3−^ sub-component. Inset: The ν_2_ PO_4_^3−^ and ν_4_ PO_4_^3−^ bands. Sub-components of the ν_2−_, ν_3−_ and ν_4_ PO_4_^3−^ bands are labelled *a*: 1047 cm^−1^, *b*: 1076 cm^−1^, *c*: 428 cm^−1^, *d*: 450 cm^−1^, *e*: 579 cm^−1^, *f*: 590 cm^−1^, *g*: 607 cm^−1^, and *h*: 614 cm^−1^.

The HAp fibers were 3–15 µm thick (Fig. 1a) and carbonate-free (Fig. 1b), since characteristic Raman peaks attributable to CO_3_^2–^ ions in calcium phosphates or calcium carbonates were not detected.^16^ The symmetric stretching mode, ν_1_ PO_4_^3–^, was narrow (FWHM = 3.25 cm^–1^) and decreased in intensity by 68 ± 10% (*p* = 0.012) between polarization configurations, i.e., depolarization ratio of 0.32. The ν_2_ PO_4_^3–^ band consists of sub-components at 428 cm^–1^ and 450 cm^–1^, of which the *∼*428 cm^–1^ sub-component appears least sensitive to polarization in terms of position, intensity, and shape. The ν_4_ PO_4_^3–^ band is comprised of sub-components at 579–580 cm^–1^, 590 cm^–1^ with a shoulder at 587 cm^–1^, 607 cm^–1^, and 614 cm^–1^. In comparison, the inorganic phase of bovine cortical bone exhibits a broader ν_1_ PO_4_^3–^ peak (FWHM = *∼*17 cm^–1^), attributable to the poor crystallinity of bone mineral, and a strong ν_1_ CO_3_^2–^ band at *∼*1072 cm^–1^. The ν_1_ CO_3_^2–^/ν_1_ PO_4_^3–^ intensity ratio (*∼*0.18) does to change between polarization configurations. In addition, a wide shoulder is present in the 1025–1055 cm^–1^ range corresponding to the ν_3_ PO_4_^3–^ band. The individual sub-components of the ν_2_ PO_4_^3–^ and ν_4_ PO_4_^3–^ bands are poorly resolved.

**Figure 2.**
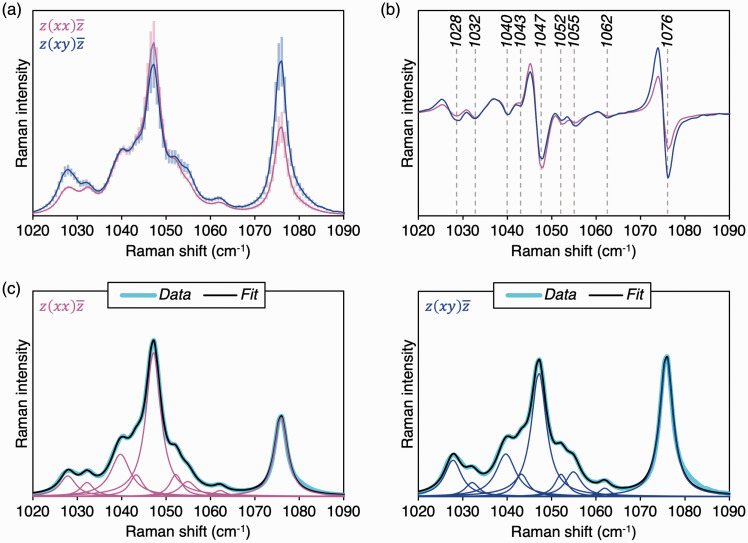
(a) The ν_3_ PO_4_^3–^ band in $\text{z}(\text{xx})\bar{\text{z}}$ (magenta) and $\text{z}(\text{xy})\bar{\text{z}}$ (blue) configurations. Average Raman spectra (normalized to the 428 cm^–1^ sub-component) and standard deviations are shown. (b) Second-derivative spectra. (c) Lorentzian curve-fitting.

The profile of the ν_3_ PO_4_^3–^ mode of synthetic HAp fibers varies between scattering configurations. Second-derivative spectra reveal nine distinct inflection points in the 1020–1090 cm^–1^ range (Fig. 2). Previously, Awonusi et al.^8^ have predicted six sub-components at 1029, 1040, 1047, 1053, 1062, and 1076 cm^–1^. However, each of the features at 1029, 1040, and 1053 cm^–1^ are found to resolve into two sub-components: 1028 cm^–1^ and 1032 cm^–1^, 1040 cm^–1^ and 1043 cm^–1^, and 1053 cm^–1^ and 1055 cm^–1^, respectively.

**Figure 3.**
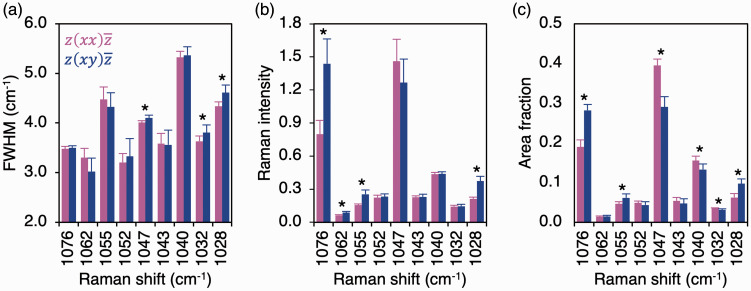
Polarization affects ν_3_ PO_4_^3–^ sub-component (a) FWHM, (b) intensity, and (c) area fraction. Wilcoxon signed-rank test (*n* = 8). Asterisk (*) denotes statistically significant difference between $\text{z}(\text{xx})\bar{\text{z}}$ and $\text{z}(\text{xy})\bar{\text{z}}$ configurations.

**Figure 4.**
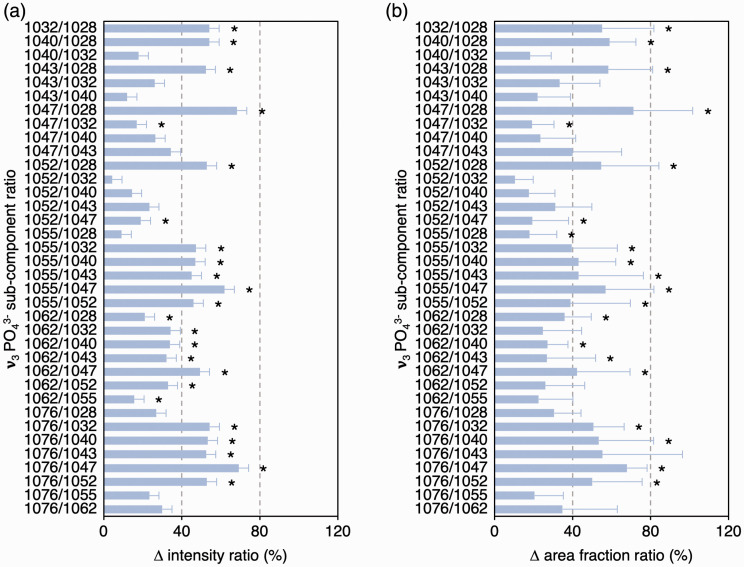
Polarization affects ν_3_ PO_4_^3–^ sub-component (a) intensity ratio and (b) area fraction ratios. Wilcoxon signed-rank test (*n* = 8). Asterisk (*) denotes statistically significant difference between $\text{z}(\text{xx})\bar{\text{z}}$ and $\text{z}(\text{xy})\bar{\text{z}}$ configurations.

Between scattering configurations, ν_3_ PO_4_^3–^ sub-component positions remained consistent, while FWHM, intensities, and area fractions of several sub-components vary (Fig. 3), which alters the intensity ratios and area fraction ratios between individual sub-components. It is, therefore, demonstrated that ν_3_ PO_4_^3–^ band profile/shape is not conserved between polarization configurations and that the individual sub-components exhibit dissimilar levels of polarization dependency. Of particular significance is the relationship between 1076 cm^–1^ and 1047 cm^–1^ sub-components, which are two of the strongest features of the ν_3_ PO_4_^3–^ band. Attributable to increases in 1076 cm^–1^ sub-component intensity and area fraction, the 1076 cm^–1^/1047 cm^–1^ ratios differ by ∼68–69% (*p* = 0.012) between the polarization configurations (Fig. 4).

Biological apatites contain varying amounts of CO_3_^2–^. The CO_3_^2–^ content of bone is taken as an indicator of bone maturation and turnover,^7,17^ and considerable attention is given to the carbonate-to-phosphate ratios of bone affected by compromised systemic conditions^18–20^ and bone surrounding implant biomaterials.^21,22^ It is likely that the 1076 cm^–1^ sub-component of the ν_3_ PO_4_^3–^ band contributes to the ν_1_ CO_3_^2–^ band, but remains a challenge to deconvolute. The scattering configuration dependency of the 1076 cm^–1^/1047 cm^–1^ ratio is also noteworthy. The contribution/intensity of the 1076 cm^–1^ sub-component may vary as a function of local, sub-micrometer/nanoscale orientation of bone mineral, thereby skewing the shape, intensity, and/or integral area of the ν_1_ CO_3_^2–^ band, thereby compromising the accuracy of carbonate-to-phosphate ratios in B-type CO_3_^2–^ substituted apatite.

## Conclusion

The ν_3_ PO_4_^3–^ band in carbonate-free HAp fibers resolves into at least nine sub-components in the 1020–1090 cm^–1^ spectral range. Sub-component FWHM, intensities, area fractions, and intensity and area fraction ratios vary between $\text{z}(\mathrm{xx})\bar{\text{z}}$ and $\text{z}(\mathrm{xy})\bar{\text{z}}$ configurations. The sub-components at 1028, 1032, 1047, 1055, and 1076 cm^–1^ exhibit pronounced sensitivity to the polarization configuration. A noteworthy change is in the 1076 cm^–1^/1047 cm^–1^ ratio between parallel and perpendicular polarized configurations. It is likely that the 1000–1100 cm^–1^ range of B-type carbonated apatites, containing the ν_1_ CO_3_^2–^ band, also includes a small contribution of the 1076 cm^–1^ sub-component of the ν_3_ PO_4_^3–^ band, which may vary as a function of local, sub-micrometer/nanoscale orientation of bone mineral, thereby skewing the shape, intensity, and/or integral area of the ν_1_ CO_3_^2–^ band, thereby compromising the accuracy of carbonate-to-phosphate ratios in B-type CO_3_^2–^ substituted apatite. For this reason, carbonate-to-phosphate ratios determined using Raman spectroscopy must be interpreted with caution.
